# The Epithelial-Immune Crosstalk in Pulmonary Fibrosis

**DOI:** 10.3389/fimmu.2021.631235

**Published:** 2021-05-19

**Authors:** Thomas Planté-Bordeneuve, Charles Pilette, Antoine Froidure

**Affiliations:** ^1^ Pôle de pneumologie, O.R.L. et dermatologie, Institut de Recherche Expérimentale et Clinique, Université catholique de Louvain, Bruxelles, Belgium; ^2^ Service de pneumologie, Cliniques universitaires Saint-Luc, Bruxelles, Belgium

**Keywords:** lung fibrosis, mucosal immunity, epithelium, mucus, innate immunity

## Abstract

Interactions between the lung epithelium and the immune system involve a tight regulation to prevent inappropriate reactions and have been connected to several pulmonary diseases. Although the distal lung epithelium and local immunity have been implicated in the pathogenesis and disease course of idiopathic pulmonary fibrosis (IPF), consequences of their abnormal interplay remain less well known. Recent data suggests a two-way process, as illustrated by the influence of epithelial-derived periplakin on the immune landscape or the effect of macrophage-derived IL-17B on epithelial cells. Additionally, damage associated molecular patterns (DAMPs), released by damaged or dying (epithelial) cells, are augmented in IPF. Next to “sterile inflammation”, pathogen-associated molecular patterns (PAMPs) are increased in IPF and have been linked with lung fibrosis, while outer membrane vesicles from bacteria are able to influence epithelial-macrophage crosstalk. Finally, the advent of high-throughput technologies such as microbiome-sequencing has allowed for the identification of a disease-specific microbial environment. In this review, we propose to discuss how the interplays between the altered distal airway and alveolar epithelium, the lung microbiome and immune cells may shape a pro-fibrotic environment. More specifically, it will highlight DAMPs-PAMPs pathways and the specificities of the IPF lung microbiome while discussing recent elements suggesting abnormal mucosal immunity in pulmonary fibrosis.

## Introduction

The role of the immune system in the development and disease course of idiopathic pulmonary fibrosis (IPF) has been a matter of heated debate over the last decades. Initial observations of increased neutrophil counts in the broncho-alveolar lavage (BAL) (1, 2) alongside the histologic presence of neutrophils, lymphocytes and macrophages in the proximity of fibrotic areas (1) led to the hypothesis that IPF starts as an inflammatory alveolitis and progresses to alveolar septal fibrosis over time. These observations formed the basis for the use of immunosuppressive therapies, in particular corticosteroids, in IPF. Although randomized controlled trials evaluating the role of steroids were missing (3, 4), observational data suggested a heterogeneous response in patients (5). In the early 2000s, the influence of immunity and immunomodulatory medication in IPF began to be questioned, with the emergence of alveolar epithelial dysfunction as one of the main contributors to pathogenesis (6) and the observations that, with further refinement of disease classification criteria (7), better characterized patients with a usual interstitial pneumonia pattern (UIP) displayed only mild inflammation (8). Finally, a milestone study assessing the effect of N-acetylcysteine, azathioprine, and prednisone in IPF reported a deleterious effect of this combination therapy (9) further weakening the “inflammatory hypothesis” in IPF. The emergence of high-throughput technologies, such as single-cell RNA sequencing, have allowed for the discovery of fibrosis-specific cell populations and fueled a renewed interest for the immune system in this disease. Thus, the place of immunity and inflammation in the course of this pathology has evolved, from causal to modulating (10) and unravelling the subtleties underlying this influence could help discover new targets and understand why immunosuppressive interventions have failed in the past.

**Figure 1:**
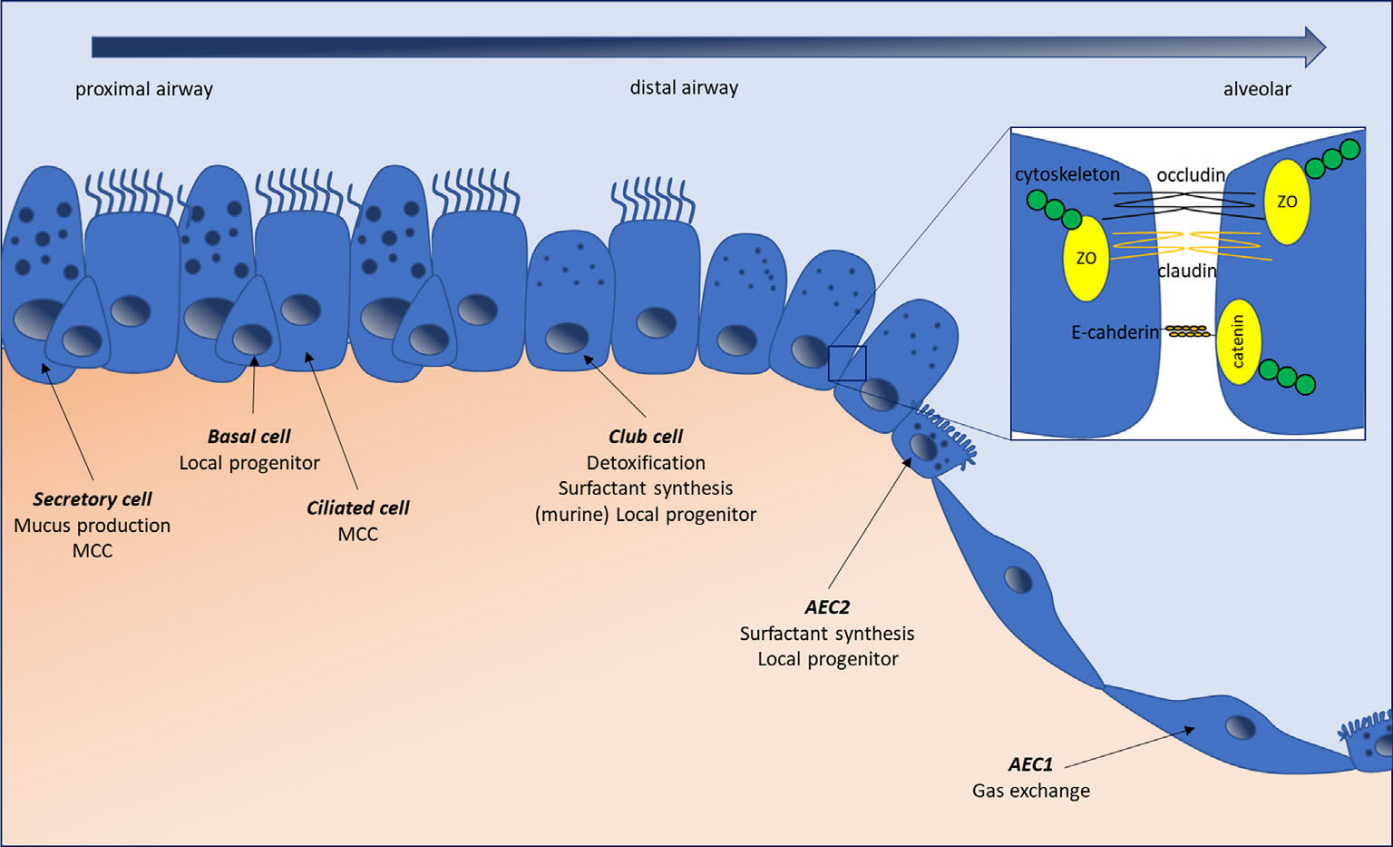
The normal lung epithelium composition changes along the respiratory tree from proximal airways to alveolar areas. Secretory cells produce the mucus lining the airways, which is moved upstream by the ciliated beats originating from ciliated cells. Basal cells have a local progenitor function, possessing the ability to differentiate into several cell types, including secretory and ciliated cells. In small airways, basal and secretory cells are progressively replaced by club (ex-Clara) cells, which can serve as local facultative progenitors (besides basal cells), secrete components of the bronchiolar lining fluid, and play a detoxifying role through their expression of cytochrome p450. In the alveoli, alveolar type-1 epithelial cells (AEC1) are responsible for gas exchange, while alveolar type-2 epithelial cells (AEC2) produce surfactant and serve as local progenitors. Epithelial cells are connected by tight- and adherens junctions, forming a continuous layer separating the intra-luminal content from the submucosal environment and regulating intercellular permeability. Tight junctions are composed of integral membrane proteins like claudins and occludins, which are linked to the cytoskeleton through cytosolic protein complexes such as Zonula Occludens (ZO). Adherens junctions, formed by E-cadherin proteins, linked to the cytoskeletion by catenins are responsible for the maintenance of cell-cell adhesion while being involved in many intracellular signaling and transcriptional pathways. MCC, mucociliary clearance.

The distal lung epithelium forms a continuous layer of cells responsible for gas transport and exchange as well as host defense. A complete overview of pulmonary cell composition can be found in (11, 12). Briefly, whereas in proximal conducting airways, it is principally composed of ciliated, secretory and basal stem cells, monostratified type-1 and type-2 alveolar epithelial cells (AEC) are present in the alveoli (11) (**Figure 1**). As the lung lays at the interface between host and environment, constantly exposed to external stimulation, a tight regulation of inflammatory mechanisms is required to preclude inadequate immune reactions. Lung epithelial cells participate in this equilibrium through several mechanisms. While the contribution of myeloid cells to lung immune mechanisms and secondary fibrosis in IPF has been extensively studied, the participation of the epithelium remains to be fully determined. Although *ex vivo* epithelial cultures are a tedious process, notably hampered by the rapid dedifferentiation of, for example, monocultured alveolar type-2 epithelial cells (AEC2) (13), both *in vivo* and *in vitro* evidence point towards the implication of the epithelium in the aforementioned processes. In this review, we will summarize how epithelial cells’ biology and their crosstalk with immune cells and microbes may, under some circumstances, conduct to aberrant, pro-fibrotic signaling in the lung. We will discuss how epithelial cells form a physical barrier through their secretion and removal of mucus, while forming a continuous cell layer, and how alterations in these mechanisms can fuel pro-fibrotic mechanisms. Furthermore, we will review the data regarding their ability to sense and react to danger and pathogen associated molecules and the existing links between alterations in those mechanisms and lung fibrosis. Finally, we will address the epithelial capacity to modulate lung immune responses, notably through the secretion of several soluble mediators (14, 15), and to trigger the recruitment, polarization and activation of pro-fibrotic myeloid cells.

## The Epithelium as a Physical Barrier

### Mucins and Mucociliary Clearance

The mucus layer covering the respiratory tract epithelium is able to trap and remove noxious stimuli thanks to mucociliary clearance and cough, forming the lung’s first line of defense in the airways (16). Mucins are glycosylated proteins that help constitute this visco-elastic layer, isolating the underlying structures from the outer world. The human lung expresses 16 different types of mucins, which can be separated into two families, namely secreted (predominantly MUC5AC and MUC5B) and membrane-bound mucins (mainly MUC1, MUC4 and MUC16) (17). Mucins fulfill multiple roles, forming a mesh hampering epithelial access to noxious stimuli, acting as lubricant as well as (decoy) receptors for pathogens, associating with several cytokines and growth factors, and, for membrane bound mucins, influencing intracellular signaling pathways such as NFκB or β-catenin (18–22). Mucin expression is regulated by numerous signals, including cytokines such as TNF-α, IL-1β, IL-6, IL-13 or IL-17, growth factors like EGF, Damage-Associated Molecular Patterns, bacterial and viral products or proteases (23–28). Of note, membrane-bound mucins consist of 2 non-covalently linked α- and β-chains, which, when exposed to physical stress, inflammatory mediators or changes in their ionic environment, can separate, causing the release of the α-chain (29).

Mucins seem to play a favoring role in the development of lung fibrosis and its subsequent course. Indeed, the most important genetic risk factor associated with IPF is the single nucleotide polymorphism (SNP) rs35705950 in the promoter region of *MUC5B* (30). This common allelic variant, present in 38% of IPF patients and 9% of controls (30), is both predictive and prognostic in lung fibrosis (31), as it is associated with a significant increase in the risk of having pulmonary fibrosis in the Framingham Heart Study population (32) and decreased mortality in 2 IPF cohorts (33). This polymorphism is linked with an increased expression of *MUC5B* (30) and its homonymous mucin protein (34). Furthermore, independently of their genetic background, IPF patients display increased levels of MUC5B in the distal airways (35, 36) and MUC5B is the main mucin present in honeycomb cysts (36). How MUC5B accumulation influences lung fibrosis is still not completely determined but could involve decreased mucociliary clearance with local inflammation or abnormal epithelialization. Supporting the former, a recent link between C3, a component of the complement cascade, the MUC5B polymorphism and IPF has been described (37). Additionally, distal overexpression of MUC5B in mice leads to a thickened mucus layer, impaired mucociliary clearance, augmented honeycomb cyst size and increased fibrosis after bleomycin challenge (38, 39). *In vivo* data indicates a crucial role for MUC5B in the maintenance of healthy interactions between the host and bacteria, as *Muc5b^-/-^* but not *Muc5ac^-/-^* animals display impaired survival related to respiratory infections (40). Impaired mucociliary clearance, present in both *Muc5b^-/-^* and Muc5b overexpressing animals could result in suboptimal clearance of organisms and increased epithelial-bacterial contact. Besides, although currently no causal relationship can be established, IPF subjects with increased bacterial loads display worse survival (41) while the presence of the rs35705950 SNP is associated with lower bacterial burden (41) and improved outcomes (33). Much less is known about the potential implication of MUC5AC in IPF. Recently, a single nucleotide variant in *MUC5AC* was described (42), but the exact effects on protein expression and clinical outcome remain to be determined. Independently from this observation and similarly to MUC5B, MUC5AC expression is increased in the distal IPF lung (36) and is expressed within HC, albeit at a much lower level (35, 36). Similarly to their secreted counterparts, the expression of MUC1 and MUC4 is increased in IPF lungs (43, 44). These mucins are involved in lung fibrosis through their α- and β-chain. In fact, the MUC1 and MUC16 extracellular domains contain the KL-6 and CA125 epitopes respectively, which have been linked with disease progression (45, 46). Furthermore, KL-6 can promote fibroblast proliferation and migration while exerting anti-apoptotic activities (47, 48) and was implicated in an *in vivo* experimental model of lung fibrosis (49). Finally, implication of the cytoplasmic tails of both MUC1 and MUC4 is suggested by the fact that their genetic and pharmacologic modulation is sufficient to protect bleomycin treated mice and by their role in TGF-β1-induced EMT or myofibroblast differentiation (43, 44).

### Intercellular Junctions

Tight junctions (TJ) and adherens junctions (AJ) act as apical junctional complexes, connecting adjacent cells, regulating the transport of solutes, allowing cell polarity and permitting the separation of the airway lumen and the underlying mucosa through a physical barrier (50, 51). Briefly, TJ are composed of integral membrane proteins, such as claudins and occludins and cytosolic protein complexes comprising Zonula Occludens proteins (ZO-1, 2, 3) (52) linked to actin binding proteins and the cytoskeleton (**Figure 1**) (51, 53). Claudin expression varies in function of the tissue (54) and these proteins can be divided in two groups based on their permeability properties, with claudins-2, -7, -10, -15 and -16 promoting paracellular flux, while claudins-1, -4, -5, -8, -11, -14 and -18 have a sealing function (55, 56). Within the human lung, claudin expression is variable, the main bronchiolar claudins being claudin-1, -2, -3, -4, -5 and -7, while alveolar cells are positive for claudin-3, -4, -7 and -18 (57–59), suggesting tailored expression in function of the localization. AJ are especially important for the maintenance of cell-cell adhesion but are also involved in many intracellular signaling and transcriptional pathways. In the alveolar epithelium, the hallmark structure of AJ consists of a complex formed by the E-cadherin cell adhesion molecules linked to the actin cytoskeleton thanks to catenins (**Figure 1**) (51). β-catenin, in particular, serves important signaling functions, linking structural junctions with the Wnt pathway. At last, desmosomes, specialized membrane complexes, help maintain the mechanical integrity of tissues and are particularly represented in tissues undergoing high mechanical stress, such as the lungs (60). They are composed by desmosomal cadherins, Armadillo proteins and plakins, and are present throughout the bronchial and alveolar epithelium (61). Lungs of patients affected by IPF present several signs of epithelial integrity disruption, with basement membrane denudation (62) and downregulation of several junctional proteins, suggesting that alterations in one, or several, of these structures are present.

Tight junctions are altered in IPF, with immunohistochemical observations showing an increased expression of occludin, claudin-1, -2, -3 and -7 and a downregulation of claudin-18 within regions of abnormal epithelialization (57–59). Discrepant results exist for claudin-4, with reports of increased (58, 59) or decreased expression (57) but this can at least partly be explained by differences in epithelial classification between studies, since alveolar and bronchiolar zones were not always separated. Measures of lung epithelial permeability through 99m-labelled diethylenetriamine penta-acetic acid (^99m^Tc-DTPA) measurement, although quite non-specific, shows that patients have faster clearance than control subjects, suggesting increased epithelial permeability (63). Similarly, intraperitoneal bleomycin injections, resulting in lung fibrosis, lead to decreased pulmonary expression of claudin-5 and -18 as well as occludins (64) while claudin-4 is upregulated after experimental acute lung injury (65). The mechanisms underlying these alterations are unclear; however, TGF-β1, one of the main profibrotic cytokines involved in IPF, is capable of inducing TJ disassembly (64), increases claudin-4 (66) and decreases claudin-18 expression (67). Interestingly, genetic deletion of *cldn18* results in (pathologic) epithelial regeneration efforts with alveolar enlargement, impaired barrier function, alveolar type-1 epithelial cell (AEC1) injury, AEC2 expansion and YAP activation, a proliferation/differentiation protein activated in IPF alveolar cells (68–70). Furthermore, preserved epithelial barrier integrity and polarization permit modulation of the interaction between growth factors or cytokines and their receptors, further implicating TJ in innate immune processes and epithelialization. For instance, expression of heregulin, a Human Epidermal growth Receptor (HER) ligand, is normally restricted to the apical surface of the lung epithelium, separated from its coreceptor HER2/3 at the basal level by intact TJ (71). Upon disruption of TJ integrity, the ligand is able to gain access to its receptor, prompting downstream signaling implicated in experimental pulmonary fibrosis (72). Although these lines of evidence point towards a role for TJ dysfunction in lung fibrosis, it is still uncertain whether TJ alterations can directly influence this process or are mere bystanders of abnormal epithelialization, necessitating further mechanistic studies before definitive conclusions can be drawn.

Loss of E-cadherin and gain of N-cadherin is a salient feature of epithelial-mesenchymal transition (EMT), a process by which epithelial cells gain mesenchymal characteristics, as observed in IPF. Accordingly, the IPF lung epithelium displays alterations in the expression of these AJ proteins, with decreased basal cell expression of E-cadherin and co-expression of E-cadherin and N-cadherin in hyperplasic pneumocytes (73). Additionally, treatment with bleomycin, either in experimental models of lung fibrosis or on an alveolar epithelial cell-line reduces E-cadherin expression (74, 75). Similarly to TJ, TGF-β1 seems to be one of the main mediators of AJ alteration, as it has the ability to downregulate E-cadherin (76, 77). A complete overview of the role of EMT in IPF is proposed by Salton et al. (78). Finally, lung-specific deletion of E-cadherin in mice results in loss of airway epithelial cells, epithelial denudation, and increased presence of α-smooth muscle actin (α-SMA) expressing cells alongside increased alveolar diameters (79).

Periplakin and desmoplakin, two plakins linking the desmosomal plaque with intermediate filaments have also been implicated in lung fibrosis. Recently, variants of *DSP*, the gene coding for desmoplakin, were associated with IPF while mRNA levels are elevated in diseased lungs (80). Periplakin was initially identified as a potential contributor to pulmonary fibrosis due to the presence of anti-periplakin antibodies in the serum of 40% of IPF patients, and alterations in its alveolar expression (61). Further mechanistic insights show that these antibodies impact epithelial migration and wound closure while BAL of IPF patients downregulates *Ppl* mRNA in murine alveolar cells (61, 81). Furthermore, *Ppl^-/^* ^-^ animals are protected from experimental lung fibrosis, display altered downstream signaling in pro-fibrotic pathway synchronously to an anti-inflammatory alveolar environment and decreased, pro-fibrotic, alternatively activated macrophages (81). No alterations of other cell junctional components could be observed, arguing against a loss of epithelial integrity and for a direct role of periplakin as modulator of its immune milieu and downstream profibrotic signals.

## The Lung Epithelium Senses and Reacts to Danger Signals

Aside from disrupting the physical barrier separating the basal membrane and submucosal tissue from the luminal content, epithelial injury also leads to the release of danger signals, so called Damage-Associated Molecular Patterns (DAMPs). This results in the activation of inflammatory pathways and the promotion of damaged structures clearance in a process of “sterile inflammation” (82). A wide variety of proteins can act as DAMPs, sharing the feature of being either mislocalized or altered. High Motility Group Box 1 (HMGB1) is the first described DAMP following the “danger theory” (83) and is normally spatially restricted to the nucleus, where it regulates DNA organization and transcription, but can act as a strong pro-inflammatory stimulus when passively released in the surrounding milieu by necro(pto)tic cells (83). Next to passive release, HMGB1 can also be actively secreted by non-necrotic cells of the immune system and intestinal epithelial cells after immune stimulation (84, 85). Similarly, the production of hyaluronan fragments from extracellular matrix high-molecular weight (HMW) hyaluronan can trigger inflammatory pathways (86). Furthermore, disruption of physical defense mechanisms will also lead to increased contact with bacterial and viral products named Pathogen-Associated Molecular Patterns (PAMPs), such as lipopolysaccharides, ds/ssRNA or unmethylated CpG DNA (87). Both DAMPs and PAMPs downstream signaling is mediated through Pattern Recognition Receptors (PRR), intracytoplasmic and membrane receptors consisting of 4 classes, Toll-Like Receptors (TLR), NOD-Like Receptors (NLR), C-type Lectin Receptors (CLR) and RIG-I-Like receptors (RLR) (88). These receptors are present on cells from the immune system, but also expressed by lung epithelial cells (15) and can trigger a wide array of effects, resulting in activation of NFκB, MAPK and interferon pathways.

### The Epithelium as Source and Target of DAMPs in IPF

Although DAMPs primarily serve an inflammatory function, they are increased in IPF and, based on experimental results, seem to be involved in fibrogenesis. As stated previously, DAMPs can originate from necro(pto)tic cells, and increased levels of RIPK3, a regulator of necroptosis have been observed in IPF lungs and in experimental, bleomycin-induced pulmonary fibrosis, particularly within alveolar epithelial cells (89). Further implication comes from the observation that HMGB1, uric acid or extracellular ATP (eATP), all recognized DAMPs, are increased in both human BAL as well as *in vivo* and *in vitro* experimental conditions (89–93). Although the origin of these signals is multiple, distal lung epithelial cells contribute to this altered environment as they show staining for HMGB1 and bleomycin-stimulated alveolar cells produce high levels of HMGB1 and eATP (90, 93). Additionally, inhibition of HMGB1 by a neutralizing antibody, of uric acid levels by a xanthine-oxidase inhibitor and interference with eATP signaling all decrease bleomycin-induced lung fibrosis (90, 92, 93). The exact mechanisms implicating DAMPs in fibrosis are currently incompletely elucidated but include direct interactions with fibroblasts as well as epithelial cells and promotion of IL-1β production, a cytokine involved in lung fibrosis (94). Indeed, addition of HMGB1 to fibroblasts promotes cell viability and myofibroblast differentiation (90, 95) while decreased IL-1β levels are observed in bleomycin instilled mice treated with anti-HMGB1 antibodies (90). HMGB1 also influences epithelial behavior, as it enhances scratch-wound closure by AECs through the production of IL-1β and activation of TGF-β1 (96), potentially fueling frustrated repair mechanisms in the alveoli, and promotes epithelial-mesenchymal transition in bronchial cells (97). Finally, HMGB1 can shape the immune contribution to fibrosis as it prompts macrophages to produce high levels of IL-1β, which could influence collagen deposition (98) and triggers the release of chemokines such as MCP-1/CCL2 by lung epithelial cells (99), a molecule known to enhance fibrocyte recruitment (100). Additionally, epithelial cells exposed to this molecule produce higher levels of TNF-α, which has been linked with TJ disassembly (101), fibroblast apoptosis (102) and EMT (103, 104). The latter pro- and anti-fibrotic effect are mirrored by *in vivo* data, reporting both protective and promoting roles of this cytokine in lung fibrosis (105, 106). Likewise, extracellular application of ATP is able to provoke an upregulation of TGF-β1, collagen and fibronectin mRNA in cultured fibroblasts (107), increases fibroblast migration and proliferation (108) while ${\text{P}2\text{X}}_{7}^{-/-}$ mice, a knock-out model for a receptor of eATP, are protected from fibrosis and show lower IL-1β levels than control animals (93).

### PAMPs and the Lung Epithelium

PAMPs are similarly capable of influencing cell behavior and ultimately fibrosis. Lipopolysaccharides (LPS), membrane components of gram-negative bacteria, recognized by the membrane receptor TLR4, have been involved in experimental lung fibrosis (109, 110), are capable of promoting fibroblast proliferation *in vitro* (111) and induce the early secretion of IL-1β, MCP-1/CCL2 or IL-8 by AEC2 (112, 113). Additionally, bacterial and viral DNA contains hypomethylated CpG zones, and treatment of UIP lung fibroblasts and healthy peripheral monocytes with CpG oligodeoxyribonucleotides (ODN), results in increased myofibroblast as well as fibrocyte differentiation respectively (114, 115). Moreover, fibroblasts from rapidly progressive IPF patients show an enhanced susceptibility to CpG stimulation, probably due to an increased expression of its cytosolic receptor TLR9 in these subjects (115). Epithelial cells are also capable of sensing and responding to CpG, with most experiments linking CpG, lung epithelium and fibrosis conducted in the alveolar A549 cell-line. Both TLR9-dependent and -independent mechanisms could be implicated. Indeed, the induction of EMT observed after CpG treatment of alveolar cells is absent after TLR9 silencing (115) but their upregulation of CCN1, a matricellular protein with pleiotropic functions implicated in IPF and experimental lung fibrosis (116), is predominantly linked to CpG-induced endoplasmic reticulum-(ER) stress (117). Interestingly, integrin αVβ6, an epithelial cell surface receptor implicated in the activation of latent TGF-β, is simultaneously upregulated, potentially linking this with increased TGF-β1 signaling. Conversely, experiments in mice showed that addition of CpG after bleomycin instillations reduced fibrosis (118), possibly reflecting immunological species differences.

### Implication of TLR in Lung Fibrosis

Additional evidence implicating these pathways and epithelial cells arise from clinical, *in vivo*, and *in vitro* studies showing alterations of PRR in lung fibrosis. Firstly, polymorphisms affecting PRR, more specifically, TLR have been associated with IPF. In 2013, the L412F *TLR3* polymorphism was linked with respiratory decline and mortality in IPF and shown to influence fibroblast proliferation (119). The same year, SNPs in the *TOLLIP* genetic locus, resulting in lower *TOLLIP* expression levels, were associated with IPF susceptibility and for one of them disease course and mortality (120). *TOLLIP* codes for the Toll-interacting protein (TOLLIP), an inhibitory adaptor protein of downstream TLR2/4 signaling, hampering NF-κB activation (121). In addition, epithelial expression of TOLLIP is associated with resistance to *in vitro* bleomycin induced apoptosis and is locally increased in an aberrant basaloid cell population in IPF (122). Furthermore, TLR2/4 expression is increased at the epithelial level in patients with an UIP pattern (123). Secondly, evidence from knock-out experiments show differential effects in experimental lung fibrosis. TLR2 and -4 are membrane PRR recognizing DAMPs (for example HMGB1 or hyaluronan fragments) and PAMPs from Gram-positive (lipoproteins) and -negative bacteria (LPS) respectively (124). They have seemingly paradoxical effects as TLR2 alteration exerts a protective, and TLR4 alteration a promoting effect on lung fibrosis. In fact, TLR2 deficiency is associated with improved survival and decreased fibrosis in an experimental model, attenuating the pro-fibrotic T_H_2 environment (125), altering immune cell recruitment (125, 126) and diminishing IL-17 production through epithelial IL-27 production (126). TLR2 is expressed by both epithelial and immune cells, and further involvement of lung epithelial cells was shown by chimeric experiments revealing that epithelial TLR2 expression is probably the main contributor to these findings (126). Conversely, TLR4^-/-^ animals show augmented deposition of collagen in the lungs when challenged with different fibrotic stimuli (127), and display a shift towards a T_H_2 immune milieu as well as decreased autophagy, potentially impacting collagen degradation (127). Illustrating the complexity and the interplay between these different mechanisms, hyaluronan low molecular weight fragments are responsible for the production of chemokine by macrophages and redundantly signal through TLR2 and 4 whereas high molecular weight hyaluronan only need TLR4 to promote AEC regeneration and renewal (128, 129). Contradictory results, suggesting a protective role of TLR4 inhibition have also been published (110) and further studies are sorely needed to evaluate the exact contribution of each component of these complex systems.

## Modified Lung Bacterial Landscape Could Influence Epithelial Biology

Fueled by negative results of culture-based assessments, the lungs were until recently considered as a sterile environment. The advent of high throughput bacterial sequencing has allowed the identification of a diversified bacterial flora in healthy human lungs which showed modifications in chronic respiratory diseases (130). Current techniques are based on the sequencing of highly conserved genes, such as the 16S ribosomal RNA gene, to identify and quantify bacterial communities and cluster them into operational taxonomic units (OTU) (131, 132). In the healthy lung, bacterial composition resembles the oropharyngeal flora and its structure is regulated through three mechanisms, namely the amount of bacterial immigration, the rate of elimination and the reproduction rate of local bacteria (133). Architectural changes and disruption of these homeostatic pathways in disease cause the genesis of niches permitting the emergence of select bacterial populations, resulting in changes in composition and diversity of airway bacteria (134–137). The epithelium from the respiratory tract can influence the two last determinants through mucus production, mucociliary clearance, secretion of inflammatory mediators as well as alterations of the local micro-environment (133, 138).

Airway bacterial composition is altered in IPF, with patients displaying increased bacterial loads and decreased diversity (41, 139). Additionally, patients with the highest bacterial burden have a markedly worse prognosis than those with lower loads, further supporting a link between bacteria and IPF (41, 140). Of note, this correlation could not be found in a recent study evaluating chronic hypersensitivity pneumonitis patients, suggesting disease specific features (139). Prior observations had identified *Streptococcus* sp., *Prevotella* sp., and *Veillonella* sp. as the most identified bacteria in IPF lungs, questioning the association between bacterial composition and disease. Several studies have suggested a relation between certain genera and OTUs, host defense pathways (140, 141), fibroblast behavior (141) or clinical outcomes (139, 142, 143). Although descriptive, these data suggest that changes in the local bacterial landscape could lead to epithelial injury as well as influence the fibrotic and immune response. Further implication of the bacterial landscape in lung fibrosis development can be gathered from animal studies in which the flora can be controlled to express no or selected bacteria. Indeed, germ-free animals instilled with bleomycin display lower mortality (140, 144) and indices of fibrosis (144). Although this data suggests a potential role of bacteria in the development of fibrosis, studies demonstrating a causal link are scarce. In one study, macrophages exposed to outer membrane vesicles from gram-negative bacteria released IL-17B through TLR2/4 sensing, subsequently inducing the secretion of chemokines and growth factor by alveolar epithelial cells, resulting in the development of pulmonary fibrosis (144). Next to influencing immune-epithelial crosstalk, certain bacteria could directly harm the epithelium by secreting cytotoxic compounds. Indeed, streptolysin (a pore-forming cytotoxin) producing *Streptococcus* and corisin (a recently discovered cytotoxic compound) secreting *Staphylococcus* had direct effects on experimental lung fibrosis, increasing AEC2 apoptosis (145) and hampering anti-fibrotic mechanisms (146). The interactions between the microbiome, the epithelium and the immune system have just started to be unraveled and form an exciting prospect for research in the coming years. Understanding the mechanisms underlying these interactions could help to identify prognostic or therapeutic targets, especially in patients developing acute exacerbations of the disease.

## The Epithelium as a Modulator of Lung Immunity

### Epithelial Injury can Promote a T_H_2 Polarized Environment

T-helper 2 (T_H_2) lymphocytes, type 2 innate lymphoid cells (ILC2) and alternatively active macrophages (M2) shape a type 2 immune landscape and form the basis of complex crosstalk networks between epithelial, mesenchymal, innate, and adaptive immunity cells. Studies conducted in typical type 2 pathology such as asthma, have revealed a major role for the airway epithelium in the genesis and maintenance of this immune milieu (147), through the recruitment, polarization and activation of myeloid cells. This environment has been involved in mechanisms of tissue repair through TGF-β1-dependent and -independent pathways. Furthermore, studies initially conducted in *S. mansoni* infected mice allowed to show that the development of fibrosis was linked with a T_H_2 environment, involving cytokines like IL-4 and IL-13 (148). These cytokines are mainly produced by T_H_2 lymphocytes, ILC2 and macrophages. In IPF, IL-4 as well as IL-13, are elevated in the BAL of patients (149), suggesting a role in lung fibrotic processes. Congruently, overexpression of GATA3, a transcription factor implicated in T_H_2 differentiation leads to augmented lung collagen deposition (150) while animals in which IL-4 and IL-13 has been modulated, are protected from bleomycin-induced lung fibrosis (151, 152). Nonetheless, IL-13 seems to be the main fibrotic driver as on the one hand overexpression of IL-13 but not IL-4 induces spontaneous lung fibrosis (153, 154) and on the other hand *IL-13^-/-^* mice but not *IL4^-/-^* are protected from FITC-related fibrosis (155). Furthermore, IL-13 promotes fibrosis by enhancing TGF-β production by macrophages and epithelial cells, influencing TGF-β activation (154), and directly impacting myofibroblast differentiation (156). Although the bases of epithelial cell implication in type 2 immunity have been extensively studied in asthma, several links can also be established in the distal lung with regards to IPF and lung fibrosis.

First of all, epithelial cells can recruit immune cells partaking in type 2 immunity and by extension IL-13 secretion. Indeed, they can secrete chemokines such as CCL17 and CCL22, acting on T_H_2 cells and ILC2, next to the eotaxins CCL11, CCL24 and CCL26 (147). Both CCL17 and CCL22 are increased in the BAL of IPF patients as well as bleomycin treated mice and are expressed by hyperplasic (alveolar) epithelial cells (157–159). Intriguingly, CCL17 but not CCL22 inhibition leads to decreased lung collagen deposition even though they both share the same receptor, CCR4 (159). The implication of eotaxins in lung fibrosis are poorly understood, nonetheless, CCL11 is increased in experimental lung fibrosis while CCL11 deficient mice are protected and both CCL11, CCL24 and CCL26 are able to influence fibroblast behavior (160–162).

Secondly, the epithelium can influence the behavior of surrounding immune cells through the secretion of IL-25, Thymic Stromal Lymphopoietin (TSLP) or IL-33, several type-2 promoting components. IL-25 can be released by different cell types, including AEC and bronchial epithelial cells (163, 164). T-cells and ILC2, are some of the targets of this cytokine and respond by expansion and secretion of type 2 cytokines like IL-4 and IL-13 (163, 165). Its potential role in disease is suggested by the fact that IPF subjects have higher IL-25 levels in their BAL compared to controls (166). This cytokine can be involved in fibrosis by both its direct effects on fibroblasts as well as its indirect influence on IL-13-dependent fibrosis. Indeed, *in vitro* data shows a direct influence on fibroblast differentiation, cytokine and growth factor secretion (167, 168). Moreover, IL-25 overexpression is associated with perivascular fibrosis in an IL-4 and IL-13 dependent manner (169) and *IL-25^-/-^* animals are protected from *S. Mansoni* and bleomycin-induced lung fibrosis due to ILC2 related IL-13 production (166), emphasizing its upstream role in type 2 immunity mediated fibrosis.

Similarly, TSLP can be produced by a wide range of cells, including epithelial and mesenchymal cells, similarly promoting a pro-T_H_2 environment (170, 171). Staining for TSLP in IPF lungs reveals the presence of this protein in alveolar epithelial cells and fibroblasts within fibroblastic foci (172). Additionally, its concentration in the BAL of patients is significantly elevated, showing an upregulation in this disease (173). Bleomycin instillation induces the expression of TSLP in bronchial and alveolar epithelial cells, but contradictory results have been published regarding the protective character of TSLP deletion in mice (174, 175). Furthermore, stimulation of primary human fibroblasts with this cytokine results in the secretion of CCL2 and chemotaxis of monocytes to the site of injury (172) while AEC undergo EMT (176). The role of TSLP thus seems complex with seemingly contradictory *in vivo* observation and further studies are needed to evaluate its exact role in the fibrotic cascade.

After injury or necrosis, epithelial full-length IL-33 (flIL-33) will be released from the cell nucleus in the surrounding environment, where neutrophil and mast cell proteases will cleave it to its modified form (mIL-33) (177). mIL-33 binds to cells expressing its receptor, ST2, such as ILC2, T_H_2 lymphocytes, macrophages, dendritic cells or mast cells, and promotes a pro-T_H_2 environment (178). Similarly to IL-25 or TSLP, IL-33 can be found in increased concentrations in the BAL and lung tissue of IPF patients (173, 179) and is upregulated in experimental lung fibrosis (179). Both full-length and the modified form seem to be involved as addition of either recombinant protein enhances collagen deposition after bleomycin challenge (179, 180). The processes underlying this effect are ill-defined but seem to be both ST2 dependent and independent. On the one hand, flIL-33 affects lung fibrosis by modulating the innate immune landscape, directly or indirectly increasing the presence of MCP-1/CCL2, IL-6, TGF-β1 and DAMPs such as HSP70, independently of ST2, IL4 or IL-13 (179). On the other hand, mIL-33 provokes the polarization of lung macrophages, ILC2 expansion and subsequent IL-13 secretion, relying on ST2 to do so (180). Interestingly, peripheral recruitment of ST2 positive cells by IL-33 seems to be one of the prevalent factors driving this observation, as selective bone-marrow ST2 deficiency was sufficient to protect mice from bleomycin lung fibrosis (181).

Next to these cytokines, other DAMPs like HMGB1 or uric acid can promote the formation of a T_H_2 driven environment. Indeed, addition of HMGB1 enhances the expression of GATA3 by T_H_2 cells and increases the levels of IL-4 and IL-13 (182) and uric acid is implicated in the release of IL-33 and TSLP by airway epithelial cells and the production of IL-13 after respiratory syncytial virus infection (183).

Finally, a T_H_2 environment can in turn affect epithelial cell biology. Indeed, continuous exposure of bronchial cells to IL-13 results in an increase in MUC5AC production and induces collagen deposition by fibroblasts in a co-culture model (184). Additionally, IL-13 alters the integrity of the bronchial epithelial barrier by downregulating TJ (185). In the distal lung, AEC2 serve a progenitor function in the alveolar epithelium and are capable of renewing AEC1. Exposure of these cells to IL-13 results in impaired AEC1 differentiation and development of a bronchiolar transcriptomic phenotype (186) aside from increased *in vitro* apoptosis (187), potentially affecting the development of lung fibrosis.

This suggests that the lung epithelium is capable of actively and passively altering its immune environment towards a type-2 polarization and thus exert a pro-fibrotic influence through an additional mechanism. Despite the fact that overwhelming evidence exists regarding the role of type 2 immunity in lung fibrosis, these findings should be contrasted with the disappointing results of therapeutic trials of IL-13 and dual IL-4/IL-13 inhibition in IPF, which both failed to meet their therapeutic endpoints (188, 189). Arguably, these results could be explained by the fact that IL-4/IL-13 are mediators of an upstream fibrotic process of which type 2 inflammation is only one of the (redundant) aspects, resulting in the observed lack of efficacy. This is illustrated by the fact that pirfenidone, one of the two currently validated treatments of IPF with broad anti-fibrotic effects, decreases IL-4 and IL-13 concentrations in the BAL of ovalbumin challenged mice (190).

### Epithelial Cells Are Implicated in Alveolar Homeostasis and Pathologic Monocyte/Macrophage Recruitment

Alveolar macrophages (AM) are a self-renewing population of the distal lung, maintaining lung homeostasis through their role in surfactant recycling, repair following injury and tightly controlled inflammatory processes (191). To exert their many functions, macrophages can notably polarize into different subsets, namely classically activated macrophages (M1) and alternatively activated macrophages (M2). Although historically, they have been divided into two subtypes, macrophage polarization should be approached as a reversible continuum rather than a definitive dichotomic classification. Briefly, M1 macrophages are induced by LPS, IFN-γ and TNF-α, produce pro-inflammatory cytokines such as IL-1β, TNF-α, IL-12, IL-23 and promote a T_H_1 response, displaying enhanced pathogenicidal properties. M2 macrophages are promoted by TGF-β, IL-4, IL-13 and secrete pro-fibrotic chemo- or cytokines like TGF-β, PDGF, or CCL18, promoting tissue repair and immunomodulation (192, 193). Damaged AEC can release a range of signals promoting the recruitment and activation of macrophages to the site of injury, fueling a pro-inflammatory environment. In a normal response, this phase would be subsequently followed by a self-limited anti-inflammatory repair stage, characterized by M2 polarization and the production of TGF-β1 or PDGF (194). Pathologic perpetuation of these processes leads to an aberrant wound response with excessive collagen deposition and ultimately organ function impairment. AEC2 dysfunction is one of the hallmark features of IPF and *in vivo* experimental data has shown that AEC2 injury is sufficient to trigger lung fibrosis (195). Furthermore, this triggers the influx of monocyte-derived macrophages (Mo-MA) possessing a pro-fibrotic phenotype *via* an interaction with CCR2, the MCP-1 receptor (196). Accordingly, *in vivo* models have subsequently demonstrated the importance of alveolar epithelial cells MCP-1/CCL2 secretion in lung fibrosis (197, 198). MCP-1/CCL2 is a chemotactic factor for myeloid cells such as monocytes, macrophages and fibrocytes (198, 199), which can also influence fibrocyte as well as fibroblast migration, proliferation, and differentiation *in vitro* (200–202). The exact link between epithelial injury and CCL2 secretion are not fully determined, but stimulation with TGF-β1 or tunicamycin (mimicking ER-stress), 2 components implicated in AEC2 dysfunction in IPF, directly upregulate CCL2 secretion by isolated AEC2 (197). Mo-MAs can replace the native AM after depletion of this compartment, for example after bleomycin administration (203), and are one of the drivers of experimental lung fibrosis (203). In line with their monocytic origin, they express high levels of *Ccr2* mRNA (204), suggesting that CCL2 (partly) mediates the recruitment of these cells. Evidence reinforcing this interaction comes from a model in which AEC-specific deletion of CCL12 (the murine equivalent of CCL2) was able to ablate the recruitment of these cells after bleomycin challenge (197). It is unclear if this mechanism similarly mediates the recruitment of a recently discovered macrophage subpopulation in IPF (205). Of note, monocytic myeloid-derived suppressor cells (M-MDSC), a population of immunosuppressive, pro-fibrotic cells also express CCR2 (206) and emerging evidence points towards their implication in IPF (207). Furthermore, IPF patients display increased concentrations of CCL2 in their BAL (208) and immunostainings have shown a partly epithelial origin for this chemokine (209). Based on overwhelming evidence implicating CCL2/CCR2 in (experimental) pulmonary fibrosis, a trial with carlumab, an anti-CCL2 antibody was conducted in IPF. Unfortunately, no effect of this treatment could be observed, and the study was halted prematurely (210). Of note, free CCL2 levels rose in the treatment, but not the placebo group (210), suggesting the activation of compensatory mechanisms.

## Concluding Remarks

**Figure 2:**
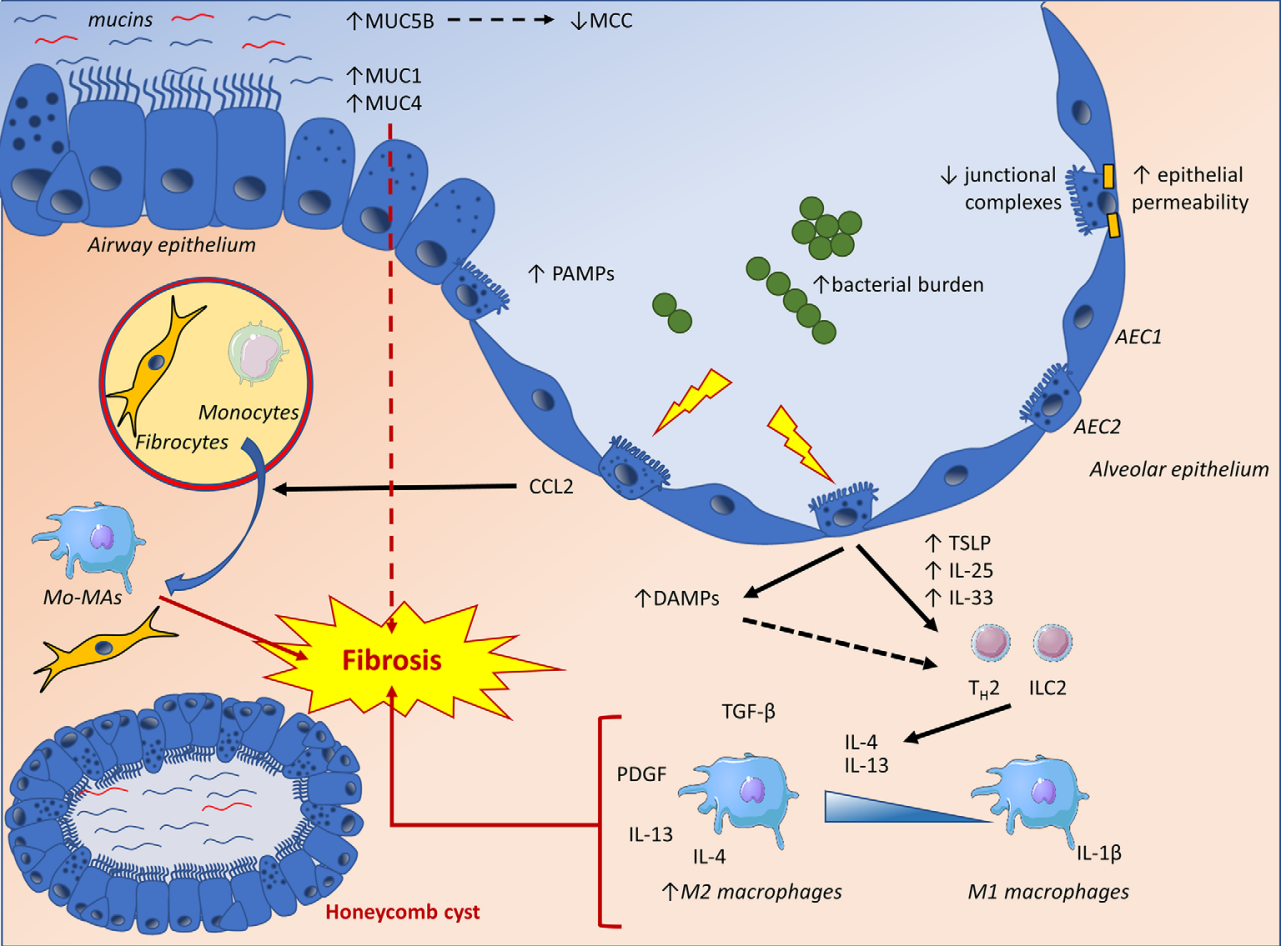
The IPF lung epithelium displays increased concentrations of secreted and membrane-bound mucins, as well as altered junctional complexes, potentially influencing local barrier mechanisms and fibrosis through impaired mucociliary clearance (MCC), promotion of epithelial to mesenchymal transition (EMT) and increased epithelial permeability. Lung epithelial cells are also confronted to an increased bacterial burden and pathogen-associated molecular patterns (PAMPs). Furthermore, epithelial damage will result in the production of damage-associated molecular patterns (DAMPs), triggering pro-inflammatory pathways and T_H_2 polarizing cytokines. These cytokines exert a pro-fibrotic influence by directly affecting mesenchymal cells and polarizing macrophages towards an alternatively activated phenotype (M2). Finally, epithelial dysfunction will result in the release of CCL2, a chemokine directly affecting fibroblasts as well as fibrocyte recruitment and differentiation while mediating the recruitment of monocytes to the site of injury. The latter will differentiate into monocyte-derived macrophages (Mo-MA), which have been implicated in lung fibrosis. AEC1, alveolar type-1 epithelial cell; AEC2, alveolar type-2 epithelial cell; Mo-MA, monocyte-derived macrophage; MCC, mucociliary clearance; ILC2, type 2 innate lymphoid cell; T_H_2, type 2 helper T-cell.

Alveolar epithelial dysfunction due to repetitive injury in susceptible/ageing lungs forms the current paradigm of IPF pathogenesis. Experimental evidence supports the involvement of the immune system in (pathologic) repair attempts and collagen deposition. The pulmonary epithelium, laying at the forefront of mucosal immunity plays a crucial role in lung homeostasis, inflammation, and subsequent repair mechanisms. It is thus capable of sensing and reacting to danger stimuli to ultimately regulate lung responses at the level of both structural and immune (myeloid) cells (**Figure 2** and **Table 1**). Aberrant alveolar epithelial biology represents a hallmark of IPF, also potentially impacting immune mechanisms. Determining the exact contribution of these mechanisms remains a challenge, as they are at the cross-point of multiple regulatory networks also involving myeloid and mesenchymal cells. For example, whether differential expression of co-stimulatory molecules such as B7 complex (including PD-L1) may interfere with the crosstalk between epithelium and immune cells remains elusive. Importantly, trials evaluating immunosuppressive medications have yielded disappointing results until now, questioning our understanding of the mechanisms at stake. Nonetheless, in-depth understanding of the epithelial contribution to the immune-fibrotic paradigm should help to appreciate the reasons underlying these clinical failures and design more targeted and effective therapies.

**Table 1:** Summary of the epithelial-immune interactions in IPF.

	Observations in IPF	Putative mechanisms (experimental data)
**Physical barrier properties**		
- **Mucus production**	↑MUC5B (35, 36) ↑MUC1 (43) ↑MUC4 (44)	MUC5B: ↓MCC (38) MUC1/4: TGF-β1 signaling, fibroblast differentiation, EMT (43, 44)
- **Intercellular junctions**	↑claudin-1, -2, -3, -7 (57–59) ↓claudin-18 (57) ↓E-cadherin (73)	↑epithelial dysfunction (69, 70) ↑polarized receptor-ligand interactions (71) ↑epithelial denudation (79)
**Environmental sensing**		
- **PRR** - **DAMPs** - **Bacterial PAMPs**	↑TLR2 ↑TLR4 ↑TLR9 (123, 211) ↑HMGB1 ↑eATP ↑uric acid ↑HA (90–93, 212) ↑Bacterial load ↓diversity (41)	TLR2^-/-^: ↓fibrosis, ↓T_H_2 environment, altered immune cell recruitment, ↓IL-17 production (125, 126) TLR4^-/-^: ↓↑fibrosis, ↑T_H_2 environment, ↓autophagy ↓AEC2 proliferation (110, 127, 128) TLR9: ↑EMT ↑myofibroblast differentiation (114, 115) ↑EMT ↑IL-1β ↑CCL2 ↑fibroblast proliferation and differentiation (90, 95, 97–99, 108) LPS: ↑fibroblast proliferation (111) ↑IL-1β ↑CCL2 (112)
**Modulation of the immune environment**		
- **T_H_2 environment promotion**	↑CCL17 ↑CCL22 ↑IL25 ↑TSLP ↑IL-33 (158, 166, 173)	↑Recruitment of T_H_2 and ILC2, ↑fibroblast proliferation, differentiation and collagen synthesis, ↑EMT (167, 176, 180)
- **Recruitment of myeloid cells**	↑CCL2 (208)	↑Recruitment of Mo-AM (197)

MCC, mucociliary clearance; EMT, epithelial-mesenchymal transition; eATP, extracellular ATP; HA, hyaluronan; LPS, lipopolysaccharide; TSLP, Thymic Stromal Lymphopoietin; Mo-AM, monocyte-derived alveolar macrophages.

## Author Contributions

TP-B designed and wrote the manuscript. CP wrote and revised the manuscript. AF designed, wrote and revised the manuscript. All authors contributed to the article and approved the submitted version.

## Funding

TP-B is the recipient of a Fonds de la Recherche Scientifique grant (FNRS grant n°ASP/DM A814). CP is postdoctoral specialist of the Fonds de la Recherche Scientifique (FNRS grants 1.R016.16 and 1.R016.18). AF is supported by the Fonds de Recherche Clinique, Cliniques universitaires Saint-Luc, as well as by the Fonds de la Recherche Scientifique (FNRS CDR grant n° J007820).

## Conflict of Interest

The authors declare that the research was conducted in the absence of any commercial or financial relationships that could be construed as a potential conflict of interest.
